# Small Vessel Disease Phenotype Associated With Monoallelic *NOTCH3* Loss-of-Function Variants

**DOI:** 10.1212/WNL.0000000000214021

**Published:** 2025-08-29

**Authors:** Josephine S. van Asbeck, Gido Gravesteijn, Minne N. Cerfontaine, Jeroen P. Vreijling, Aat A. Mulder, Roman I. Koning, Mark C. Kruit, Anna Bersano, Keith W. Muir, Sam J. Neilson, James F. Meschia, Md Manjurul Islam Shourav, Andreas Koupparis, Yi-Chung Lee, Nicola Rifino, Remco J. Hack, Esther A.R. Nibbeling, Saskia A.J. Lesnik Oberstein, Julie W. Rutten

**Affiliations:** 1 Department of Clinical Genetics, Leiden University Medical Center, Leiden, the Netherlands;; 2 Electron Microscopy Facility, Department of Cell and Chemical Biology, Leiden University Medical Center, Leiden, the Netherlands;; 3 Department of Radiology, Leiden University Medical Center, Leiden, the Netherlands;; 4 Cerebrovascular Unit, Fondazione IRCCS Istituto Neurologico “Carlo Besta,” Milan, Italy;; 5 Institute of Neuroscience and Psychology, University of Glasgow, Queen Elizabeth University Hospital, Glasgow, United Kingdom;; 6 Department of Neurology, Mayo Clinic, Jacksonville, United States, FL;; 7 The Cyprus Institute of Neurology and Genetics, Cyprus, Nicosia;; 8 Department of Neurology, Taipei Veterans General Hospital, Taiwan;; 9 Department of Neurology and Brain Research Center, National Yang Ming Chiao Tung University, Taiwan; and; 10 Center for Intelligent Drug Systems and Smart Bio-devices (IDS2B), National Yang Ming Chiao Tung University, Hsinchu, Taiwan.

## Abstract

**Background and Objectives:**

Monoallelic cysteine-altering *NOTCH3* (*NOTCH3*^*cys*^) variants cause the adult-onset small vessel disease cerebral autosomal dominant arteriopathy with subcortical infarcts and leukoencephalopathy (CADASIL), and biallelic *NOTCH3* loss-of-function (*NOTCH3*^*lof*^) variants cause a rare, childhood-onset small vessel disease. Whether monoallelic *NOTCH3*^*lof*^ variants also cause a small vessel disease is subject of debate. The aim of this study was to delineate the small vessel disease phenotype of individuals with a monoallelic *NOTCH3*^*lof*^ variant and to compare it with CADASIL.

**Methods:**

In this observational study, monoallelic *NOTCH3*^*lof*^ cases were ascertained in Genome Aggregation Database (gnomAD); UK Biobank; 6 clinical centers from Europe, Asia, and the United States; and literature. In gnomAD, *NOTCH3*^*lof*^ allele frequency was determined. In UK Biobank, normalized white matter hyperintensity volume (nWMHv), peak width of skeletonized mean diffusivity (PSMD), lacune count, and stroke were compared among *NOTCH3*^*lof*^ cases, *NOTCH3*^*cys*^ cases, and controls. In clinical *NOTCH3*^*lof*^ cases, white matter hyperintensities, lacune count, and stroke incidence were assessed, and skin vessel wall pathology was analyzed using immunohistochemistry and electron microscopy.

**Results:**

In gnomAD, 306 *NOTCH3*^*lof*^ variants were identified (allele frequency 0.6/1,000). In UK Biobank, 102 *NOTCH3*^*lof*^ cases were ascertained (median age 58 years, range 40–69, 55% female). *NOTCH3*^*lof*^ cases had an increased nWMHv (Δ0.44 mm^3^, *p* < 0.001) and PSMD (Δ0.19 × 10^−4^, *p* = 0.017) compared with controls. nWMHv and PSMD in *NOTCH3*^*lof*^ cases were comparable to *NOTCH3*^*cys*^ cases; however, in contrast to *NOTCH3*^*cys*^ cases, *NOTCH3*^*lof*^ cases did not have an increased stroke risk compared with controls. Clinically ascertained *NOTCH3*^*lof*^ cases (n = 69, median age 50 years, range 20–94, 54% female) often had white matter hyperintensities (28/32, 88%) while lacunes (12/32, 38%) and stroke (11/69, 15%) were predominantly seen in cases with cardiovascular risk factors and at advanced age. Skin vessels of *NOTCH3*^*lof*^ cases more frequently showed abundant vessel wall collagen deposition compared with *NOTCH3*^*cys*^ cases and controls (37% vs 10% [*p* = 0.016] and 5% [*p* < 0.001] of vessels).

**Discussion:**

We conclude that monoallelic *NOTCH3*^*lof*^ variants cause a small vessel disease that (1) remains subclinical in most cases but may be exacerbated by cardiovascular risk factors and aging, and (2) is distinct from CADASIL regarding vessel pathology and disease severity. These findings will guide counseling and management of individuals in whom a *NOTCH3*^*lof*^ variant is found.

## Introduction

The transmembrane NOTCH3 receptor, one of the 4 mammalian NOTCH homologs, is primarily expressed by vascular smooth muscle cells, where it plays an important role in cell differentiation and maturation.^1^ A complete loss of Notch3 causes progressive dilatation and tortuosity of brain vessels in *Notch3*^−/−^ mice,^2^ and humans with biallelic *NOTCH3* loss-of-function (*NOTCH3*^*lof*^) variants present with a severe, childhood-onset small vessel disease.^3-10^ Whether monoallelic *NOTCH3*^*lof*^ variants also cause a small vessel disease is subject of debate.^3,8,10-17^

Monoallelic *NOTCH3*^*lof*^ variants (frameshift and nonsense variants that lead to degradation of the mutant transcript through nonsense-mediated RNA decay) have been described in over 50 cases to date. Half of these case reports and case series classified the variant as benign^3,8,10-12^ while the other half considered the variant causative of a small vessel disease phenotype.^13-18^ The reported neuroimaging and clinical findings vary widely, ranging from a family in which the *NOTCH3*^*lof*^ variant segregates with young adult-hood–onset stroke^18^ to an asymptomatic 50-year-old with no signs of small vessel disease on brain MRI, in whom the variant was ascertained after predictive DNA testing.^11^ In a subset of cases, electron microscopy analysis of skin vessels was performed, showing electron lucent vacuoles in the cell cytoplasm, endothelial protrusions, a thickened basal membrane, and excess collagen fibrils surrounding the vascular smooth muscle cells, although none of these was reported consistently.^3,11-13,18,19^

The most prevalent *NOTCH3*-related disorder is cerebral autosomal dominant arteriopathy with subcortical infarcts and leukoencephalopathy (CADASIL), a small vessel disease characterized by adult-onset subcortical ischemic strokes, vascular dementia, migraine with aura, and psychiatric symptoms, with a variable disease severity.^20-22^ On neuroimaging, patients with CADASIL have progressive white matter hyperintensities (WMHs), with a predilection for the anterior temporal lobes and external capsules.^23^ In later disease stages, WMHs are superimposed by lacunes, perivascular spaces (PVSs), atrophy, and microbleeds.^22,24^ CADASIL-causing *NOTCH3* variants are almost exclusively missense variants that lead to an alteration in the canonical number of 6 cysteine residues in one of the protein's 34 epidermal growth factor-like repeat (EGFr) domains (*NOTCH3*^*cys*^ variants) and can be classified as high, moderate, or low risk of developing CADASIL.^21,25^ *NOTCH3*^*cys*^ variants are associated with NOTCH3 protein aggregation in small and medium-sized arteries, which can be visualized by a positive NOTCH3 immunostaining and granular osmiophilic material (GOM) deposits in electron microscopy images.^26,27^ CADASIL mouse models and patient studies support a central role for NOTCH3 protein aggregation in CADASIL pathophysiology.^21,26,28^ *NOTCH3*^*cys*^ variants typically do not impair NOTCH3 signaling function, with the exception of variants located in the ligand-binding domain (EGFr 10–11).^28-31^ Although most patients with CADASIL have a monoallelic *NOTCH3*^*cys*^ variant, biallelic cases have been reported with a largely overlapping small vessel disease phenotype.^10^

In this study, we aimed to clarify the small vessel disease phenotype associated with monoallelic *NOTCH3*^*lof*^ variants. To this end, we characterized 69 clinically ascertained cases and 102 population-dwelling individuals with a monoallelic *NOTCH3*^*lof*^ variant, using genetic, histopathologic, neuroimaging, and clinical data, and we compared them with individuals with *NOTCH3*^*cys*^ variants and controls.

## Methods

### Study Design and Data Sources

To analyze the small vessel disease phenotype in cases with a monoallelic *NOTCH3*^*lof*^ variant, we conducted an observational retrospective study, using data from (1) the Genome Aggregation Database (gnomAD), an open-access human allele frequency reference data set that aggregates data from a variety of large-scale sequencing projects (n = 807,162 exomes)^32^; (2) UK Biobank, a prospective cohort with genetic and phenotypic data on community-dwelling individuals from the United Kingdom (n = 502,419 exomes)^33^; (3) unpublished clinical cases, ascertained in 6 hospitals from Europe, Asia, and the United States; and (4) previously published cases identified in PubMed Central.

### Standard Protocol Approvals, Registrations, and Participant Consents

Data from gnomAD v.4.1.0 are publicly available. Data on cases in UK Biobank were analyzed under UK Biobank Application Number 74162. All novel clinical cases with a *NOTCH3*^*lof*^ variant gave their consent to participate in this study. One patient from the United States was part of an IRB-approved retrospective review (the Mayo Clinic Institutional Review Board, IRB number 23-012705). Additional written consent for disclosure was obtained from the patients in the Dutch family with the *NOTCH3* c.1321C>T; p.(Arg441*) variant. This study was approved by the LUMC Review Committee Biobank & Biomaterials (reference number RP24.024).

### Ascertainment of Study Participants

In gnomAD v4.1.0 and UK Biobank, frameshift and stop-gain variants in the canonical *NOTCH3* transcript ENST00000263388 were ascertained.^32^ Variants downstream of amino acid 1955 (nucleotides 5823–5825) were excluded because these variants are predicted to escape nonsense-mediated decay and are associated with lateral meningocele syndrome.^34^ Clinical cases with a monoallelic *NOTCH3*^*lof*^ variant were identified in diagnostic genetic laboratories across 6 clinical centers in Europe, Asia, and the United States. In addition, previously published monoallelic *NOTCH3*^*lof*^ cases were identified using the following Medical Subject Headings terms in PubMed Central: Receptor, Notch3, and Loss of Function Mutation, along with variations on the search terms Monoallelic; Heterozygous; Hypomorphic; Null mutation; and Loss of function. Novel clinically ascertained cases and clinical cases identified in literature were combined into 1 clinical sample for analysis. All cases were aged 18 years and older and had a truncating *NOTCH3* variant resulting in a premature stop codon upstream of amino acid 1955 (nucleotides 5823–5825).

For each UK Biobank case with a *NOTCH3*^*lof*^ variant and available brain T2-weighted fluid-attenuated inversion recovery (FLAIR) MR images, 2 controls matched for age and cardiovascular risk factors (hypertension, dyslipidemia, diabetes type 1 or 2, smoking) were included. A second control group consisted of 42,950 individuals in UK Biobank with available brain MRI in whom *NOTCH3*^*lof*^ and *NOTCH3*^*cys*^ variants were excluded. For the comparison with individuals with CADASIL, individuals with a *NOTCH3*^cys^ variant in one of the 34 EGFr domains (amino acids 40–1,373) were ascertained in UK Biobank. *NOTCH3*^*cys*^ variants were stratified into low, moderate, or high risk based on their variant position, as previously described.^21^

### Data Collection

In gnomAD v4.1.0, the allele count for each variant was divided by the corresponding allele number to determine the total cumulative allele frequency and the cumulative allele frequency per ethnicity.

In UK Biobank, brain MRI data including T1, T2 FLAIR, and susceptibility-weighted imaging sequences (3.0T field strength) and information on volume of white matter hyperintensities, volume of the gray and white matter, estimated total intracranial volume, mutational status, sex, age, and tobacco smoking were extracted. Medication records and ICD-9, ICD-10, and noncancer illness codes were used to evaluate medical histories for hypertension, dyslipidemia, diabetes type 1 or 2, stroke, vascular dementia, psychiatric diagnoses, migraine (with and without aura), and headaches (eAppendix 1). The presence of headaches and the diagnosis of migraine (without aura) were combined into 1 outcome (“migraine or headaches”).

In the clinically ascertained cases, patient files and publications were queried for age, sex, past or current hypertension, dyslipidemia, diabetes type 1 or 2, smoking, history of stroke, cognitive impairment, migraine (with or without aura), headaches, psychiatric diagnoses, skin biopsy results, small vessel disease neuroimaging markers, and results of genetic testing.

### Neuroimaging Analysis

In cases ascertained in UK Biobank, WMH volume (WMHv) as provided by UK Biobank was divided by the total intracranial volume to calculate the normalized WMHv (nWMHv). A fully automated shell script was used to acquire the individual peak width of skeletonized mean diffusivity (PSMD).^35^ Lacunes, cerebral microbleeds, and PVSs were scored in accordance with the STRIVE-2 criteria by a researcher blinded to genotype (M.N.C.); atypical or ambiguous findings were reviewed by a second independent blinded observer (J.W.R.).^36^ PVSs were analyzed in the basal ganglia, subinsular region, global white matter, temporal lobe, occipital lobe, cerebellum, and mesencephalon according to a 4-grade semiquantitative scale as described before.^37^ Brain MRI scans of the unpublished clinical cases, either 3.0T or 1.5T field strength, were evaluated by a neuroradiologist.

### RNA Analysis, Immunohistochemistry, and Electron Microscopy

Detailed descriptions of all experimental methods are provided in eAppendix 2. In 3 unpublished clinical Dutch cases with the *NOTCH3* c.1321C>T; p.(Arg441*) variant, two 4-mm skin punch biopsies were taken from the lateral upper arm and were compared with samples of 3 individuals with a *NOTCH3*^cys^ variant (p.(Arg169Cys); p.(Arg207Cys); p.(Cys222Tyr)) and 3 controls selected from the prospective CADASIL cohort study DiViNAS.^31^ For analysis of nonsense-mediated decay, fibroblasts were cultured from the skin biopsies and fibroblast RNA was isolated and reverse transcribed. Reverse transcription–PCR on the complementary DNA (cDNA) was performed using an exon 8 forward primer and an intron-spanning exon 8–9 reverse primer (eAppendix 3). Genomic DNA was extracted from whole blood according to standard protocols and was amplified using an exon 8 forward and reverse primer. Sanger sequencing was performed on 1 μL of diluted genomic DNA and 1 μL of PCR product from the cDNA. For analysis of vascular NOTCH3 aggregation, half of the skin punch biopsy was fixed in formalin and embedded in paraffin, and skin biopsy sections were incubated with an antibody against the NOTCH3 extracellular domain (1E4, dilution 1:1,000, Millipore, Burlington, MA).^26^ Skin biopsy processing for electron microscopy analysis was performed as previously described.^38,39^ Electron microscopy images of skin blood vessels were analyzed by independent blinded observers for the presence of GOM (A.A.M., G.G., J.S.v.A.) and electron lucent vacuoles, endothelial protrusions, and collagen depositions in the basal membrane (G.G., J.S.v.A.).^3,18^

### Statistical Analysis

Numerical variables with a skewed distribution were described using median and interquartile range (IQR). Numerical variables with a normal distribution were described using mean and standard deviation. Binary categorical variables were described as the proportion of positive cases. For the analysis of the UK Biobank sample, cardiovascular risk factors (hypertension, dyslipidemia, diabetes mellitus type 1 or 2, and current or previous smoking) of each case were combined into the variable “CVRn” (defined as the number of cardiovascular risk factors present at baseline) to reduce the number of variables.^40^ nWMHv and PSMD were transformed using the natural logarithm to obtain a normal distribution. After correction for age at MRI, sex, and CVRn, linear regression models were used to compare nWMHv and PSMD among *NOTCH3*^*lof*^ cases, *NOTCH3*^*cys*^ cases, and controls from UK Biobank. nWMHv and PSMD were also compared between *NOTCH3*^*lof*^ cases and controls matched for age and cardiovascular risk factors. Logistic regression models were used to test whether the proportions of individuals with stroke, migraine or headaches, and psychiatric disorders differed among *NOTCH3*^*lof*^ cases, *NOTCH3*^*cys*^ cases, and controls after correction for age at recruitment, sex, and CVRn. In the skin biopsies, abundance of vessel wall collagen was scored as absent or present. Fisher exact tests were used to compare the proportion of vessels with an abundance of vessel wall collagen between *NOTCH3*^*lof*^ cases and controls, and between *NOTCH3*^*lof*^ cases and *NOTCH3*^*cys*^ cases (eAppendix 2). All statistical analyses were 2-sided tests with a threshold for statistical significance of 0.05, conducted using IBM SPSS Statistics software, version 25.

### Data Availability

The data that support the findings of this study are available from the corresponding author (J.W.R.), on reasonable request.

## Results

We identified 159 individuals with distinct *NOTCH3*^*lof*^ variants in gnomAD (allele count = 306), 102 cases with a monoallelic *NOTCH3*^*lof*^ variant in UK Biobank, and 69 clinically ascertained *NOTCH3*^*lof*^ cases (17 unpublished cases and 52 cases from literature).

### Frequency of *NOTCH3*^*lof*^ Variants Worldwide

In gnomAD v.4.1.0 (N = 807,162 exomes), the frequency of monoallelic *NOTCH3*^*lof*^ variants was 0.6/1,000. *NOTCH3*^*lof*^ variants were seen in individuals from all ethnicities except for those from Middle Eastern ancestry, with frequencies ranging from 0.1 per 1,000 in the African population to 1.8 per 1,000 in the Ashkenazi Jewish and South Asian populations (eAppendices 4–5). There were no individuals with biallelic *NOTCH3*^*lof*^ variants in gnomAD. The variants were located throughout the *NOTCH3* exons 1 to 32; 219 (72%) were frameshift variants, and 87 (28%) were stop-gain variants. The most frequent variant was c.112del; p.(Ala38Leufs*198), present in 47, mainly European, individuals.

### Small Vessel Disease Signs and Symptoms in UK Biobank *NOTCH3*^*lof*^ Cases

**Figure 1:**
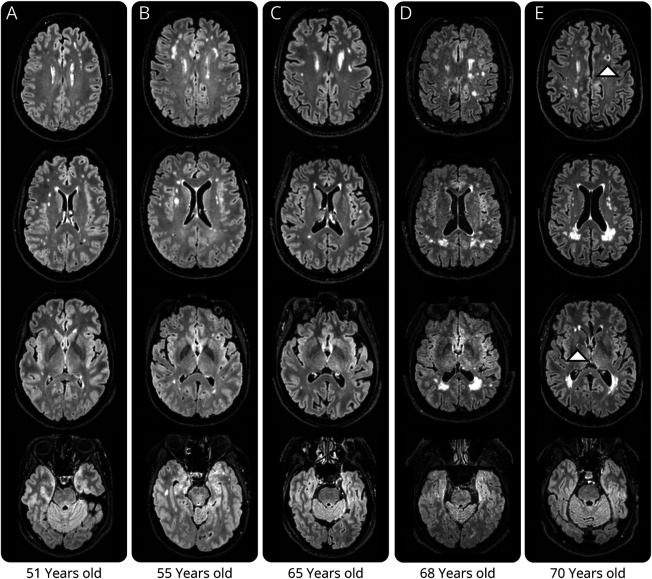
Neuroimaging Findings in UK Biobank *NOTCH3*^*lof*^ Cases (A–E) Representative brain T2 fluid-attenuated inversion recovery images illustrating the spectrum of small vessel disease neuroimaging abnormalities in UK Biobank *NOTCH3*^*lof*^ cases. All *NOTCH3*^*lof*^ cases in UK Biobank had white matter hyperintensities, 1 had lacunes, and none had microbleeds on susceptibility-weighted imaging. WMHs were typically periventricular and were present as small to medium-sized punctate foci in the deep white matter (A–C), including in the anterior temporal lobe (B), but not in the external capsules. There were 2 older *NOTCH3*^*lof*^ cases with vascular risk factors, who had confluent WMHs (D, E). One case (D) was a 68-year-old man with a history of dyslipidemia, diabetes, and smoking, and the other (E) was a 70-year-old man with a history of smoking who also had a lacune in the left frontal lobe and a lacune in the globus pallidus (arrowheads). *NOTCH3*^*lof*^ = *NOTCH3* loss-of-function; WMH = white matter hyperintensity.

**Figure 2:**
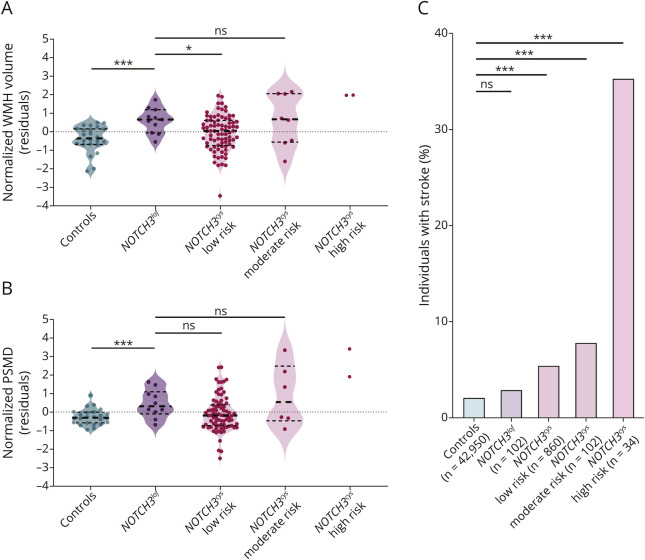
*NOTCH3*^*lof*^ Variants Are Associated With White Matter Hyperintensity Volume and PSMD, but Not With Risk of Stroke in UK Biobank (A, B) Violin plots showing the residuals of linear regression models after correction for age, sex, and cardiovascular risk factors. (A) WMH volume did not differ significantly between *NOTCH3*^*lof*^ cases and moderate-risk *NOTCH3*^*cys*^ cases (*p* = 0.981), whereas it was increased compared with low-risk *NOTCH3*^*cys*^ cases (*p* = 0.024), matched controls (*p* < 0.001), and the total of 42,950 individuals with MRI in UK Biobank (*p* < 0.001). (B) PSMD in *NOTCH3*^*lof*^ cases did not differ significantly from that in moderate-risk *NOTCH3*^*cys*^ cases (*p* = 0.568), whereas it was increased compared with matched controls (*p* < 0.001). (C) Bar charts showing the percentage of individuals with stroke per genotype. Logistic regression models did not show a difference in the risk of stroke between *NOTCH3*^*lof*^ cases and controls (OR 1.3, 95% CI 0.4–4.3, *p* = 0.636). Moderate-risk *NOTCH3*^*cys*^ cases did have an increased risk of stroke compared with controls (OR 4.4, 95% CI 2.1–9.3, *p* < 0.001). *NOTCH3*^*cys*^ = cysteine-altering *NOTCH3*; *NOTCH3*^*lof*^ = *NOTCH3* loss-of-function; ns = not significant; OR = odds ratio; PSMD = peak width of skeletonized mean diffusivity; WMH = white matter hyperintensity. Values are significant at ***p < 0.001, **p < 0.01, and *p < 0.05.

The median age of the 102 *NOTCH3*^*lof*^ cases in UK Biobank was 58 years (range 40–69 years). Brain MRI was available for 12 cases (Table 1, eAppendices 6–8). All cases with brain MRI had WMHs; these were typically periventricular and were present as small to medium-sized punctate foci in the deep white matter, including the anterior temporal lobe (Figure 1). One *NOTCH3*^*lof*^ case also had 1 WMH lesion in the cerebellum. Compared with controls matched for age, sex, and cardiovascular risk factors, *NOTCH3*^*lof*^ cases had a higher nWMHv (median 0.23 mm^3^ vs 0.67 mm^3^, IQR 0.09–0.48 vs 0.14–0.80, mean difference after transformation 1.00, 95% CI 0.48–1.52, *p* < 0.001; Figure 2A) and a higher PSMD (median 2.27 × 10^−4^ vs 2.46 × 10^−4^, IQR 2.00–2.45 × 10^−4^ vs 2.29–2.99 × 10^−4^, mean difference after transformation 0.72, 95% CI 0.16–1.28, *p* = 0.017; Figure 2B). nWMHv in *NOTCH3*^*lof*^ cases was also significantly higher compared with the 42,950 controls with MRI in UK Biobank (median 0.19 mm^3^, IQR 0.10–0.38, mean difference after transformation 1.44, 95% CI 0.85–2.03, *p* < 0.001) (eAppendix 9). nWMHv was comparable between *NOTCH3*^*lof*^ cases and 8 moderate-risk *NOTCH3*^*cys*^ cases (median 0.83 mm^3^, IQR 0.14–1.60, mean difference after transformation 0.01, 95% CI −1.23 to 1.20, *p* = 0.981). One of the 12 *NOTCH3*^*lof*^ cases with MRI had lacunes (age at MRI 70 years), whereas 3 of 8 moderate-risk *NOTCH3*^*cys*^ cases with MRI had lacunes (median age at MRI 70, range 52–72). None of the *NOTCH3*^*lof*^ cases had microbleeds, and the normalized brain volume did not differ from controls (Table 1, eAppendix 9). Three of the 102 *NOTCH3*^*lof*^ cases had a history of stroke (3%), their age at stroke was 64–68 years, and all had 1 or more cardiovascular risk factors (Table 1). After correction for age, sex, and cardiovascular risk factors, there was no significant difference in risk of stroke between the 102 *NOTCH3*^*lof*^ cases and 42,950 UK Biobank controls (2.9% vs 2.1%, odds ratio [OR] 1.3, 95% CI 0.4–4.3, *p* = 0.636, Figure 2C). Conversely, cases with a moderate-risk *NOTCH3*^*cys*^ variant did have an increased stroke risk when compared with controls (7.8%, OR 4.4, 95% CI 2.1–9.3, *p* < 0.001) (Figure 2C). *NOTCH3*^*lof*^ cases also did not have an increased risk of psychiatric disorders (9.8% vs 14.9%, OR 0.6, 95% CI 0.3–1.3, *p* = 0.196) or an increased risk of headaches (10.8% vs 9.8%, OR 1.1, 95% CI 0.6–2.2, *p* = 0.674), compared with controls (eAppendix 10). None of the *NOTCH3*^*lof*^ cases had migraine with aura, and only 1 had a diagnosis of vascular dementia: this was a 69-year-old woman with a history of diabetes and hypertension.

**Table 1:** Characteristics of *NOTCH3*^*lof*^ Cases in UK Biobank and in the Clinical Data Set

	Included data sets	
	UK Biobank (n = 102)	Clinical cases (n = 69)
Age at recruitment, y, median (range)	58 (40–69)	50 (20–94)^a^
One or more cardiovascular risk factors, n (%)	69 (68)	19 (56)^a^
Hypertension, n (%)	39 (38)	9 (26)
Dyslipidemia, n (%)	24 (24)	5 (15)
Diabetes, n (%)	5 (5)	0 (0)
Smoking, n (%)	39 (38)	8 (23)
Stroke, n (%)	3 (3)	11 (16)
Age at first stroke, y, median (range)	66 (64–68)	49 (38–78)
Cardiovascular risk factors, n (%)	3 (100)	7 (64)
Headaches or migraine without aura, n (%)	11 (11)	13 (19)
Migraine with aura, n (%)	0 (0)	6 (9)
Psychiatric diagnosis, n (%)	10 (10)	5 (7)
Whole-exome sequencing or gene panel testing, n (%)	102 (100)	5 (7)
Cases with MRI, n (%)	12 (12)	32 (45)^a^
Age at MRI, y, median (range)	65 (51–80)	54 (27–94)
White matter hyperintensities, n (%)	12 (100)	28 (88)
Lacunes present, n (%)	1 (8)	12 (38)
Age at MRI, y, median (range)	70 (N/A)	63 (39–94)
Cardiovascular risk factors, n (%)	1 (100)	10 (83)
Cerebral microbleeds present, n (%)	0 (0)	4 (13)
Age at MRI, y, median (range)	N/A	63 (58–69)
Cardiovascular risk factors, n (%)	N/A	3 (75)

Abbreviations: IQR = interquartile range; N/A = not applicable; *NOTCH3*^*lof*^ = *NOTCH3* loss-of-function.

The clinical data set consisted of 17 unpublished cases from centers in Europe, the United States, and Asia and 52 cases from literature.

a For 34 previously published monoallelic *NOTCH3^lof^* cases,^10^ no individual-level data were available for age, cardiovascular risk factors, and MRI abnormalities.

### Clinical and Neuroimaging Features in Clinically Ascertained *NOTCH3*^*lof*^ Cases

The data set of clinically ascertained *NOTCH3*^*lof*^ cases consisted of 69 individuals (median age 50 years, range 20–94; 17 unpublished cases from centers in Europe, the United States, and Asia and 52 from literature). Neuroimaging data were available for 32 cases (Table 1; eAppendix 11), of whom 28 (88%) exhibited white matter hyperintensities on brain MRI. There were 12 cases with lacunes (12/32, 38%) and 4 cases with microbleeds (4/32, 13%). Cases with lacunes or microbleeds had a median age of 63 years (range 39–94), and 85% (11/13) had 1 or multiple cardiovascular risk factors. The 13 cases without lacunes or microbleeds had a median age of 49 years (range 27–59), and 39% (5/13) had cardiovascular risk factors. Stroke was reported in 11 of 69 cases (median age 49 years, range 38–78); 7 of these also had cardiovascular risk factors (64%). Three of the 11 cases with stroke symptoms showed no signs of ischemic or hemorrhagic infarction on brain MRI (27%), although information on diffusion-weighted imaging sequences from the acute setting was not available in these cases. Migraine with aura was reported in 6 (9%), headaches in 13 (19%), and psychiatric disorders in 5 (7%) of the 69 *NOTCH3*^*lof*^ cases. In 5 of the cases, whole-exome sequencing or gene panel sequencing was performed while in the others (64/69, 93%), genetic testing was limited to (parts of) *NOTCH3*.

### Skin Blood Vessel Analysis in *NOTCH3*^*lof*^ Cases

**Figure 3:**
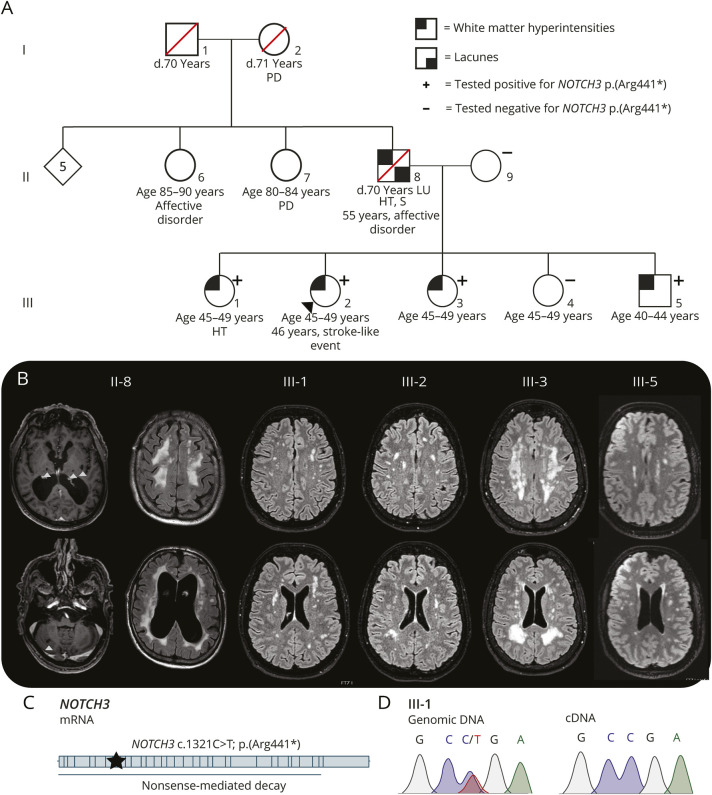
White Matter Hyperintensities and Late-Onset Lacunes in a Dutch Pedigree With a *NOTCH3*^*lof*^ Variant (A) Pedigree of a Dutch family with a *NOTCH3* c.1321C>T; p.(Arg441*) variant. The index' medical history reported a “stroke-like event” at age 46 years, without evidence of infarction on brain CT and MRI. The other siblings were asymptomatic. Except for 1 sibling with well-controlled hypertension (III-1), the siblings did not have cardiovascular risk factors. The father had a high cardiovascular risk factor burden, with over 50 pack-years of smoking, daily alcohol use, and hypertension. He was diagnosed with a normal pressure hydrocephalus, attributed to a neurotrauma at age 53 years. Up to age 67 years, he had normal scores on cognitive tests, including 30/30 on the MMSE. None of his family members had a history of stroke or dementia. (B) Representative brain T1 and T2 fluid-attenuated inversion recovery images. The father (II-8) had confluent deep white matter and periventricular white matter hyperintensities; atrophy; and 7 lacunes located in the thalamus, external capsules, and cerebellum (white arrowheads) at age 70 years. All the affected siblings had white matter hyperintensities but no other small vessel disease markers and no stroke or cognitive decline. (C) Schematic representation of the *NOTCH3* mRNA. The *NOTCH3* c.1321C>T; p.(Arg441*) variant causes a premature termination codon in exon 8. (D) Genomic DNA and primary patient fibroblast mRNA analysis. The *NOTCH3* c.1321C>T; p.(Arg441*) variant that was identified in the genomic DNA was not present in the reversed-transcribed fibroblast mRNA (cDNA), indicating degradation of the mutant allele through nonsense-mediated decay. cDNA = complementary DNA; HT = hypertension; LU = lung cancer; mRNA = messenger RNA; *NOTCH3*^*lof*^ = *NOTCH3* loss-of-function; PD = Parkinson disease; S = smoking.

**Figure 4:**
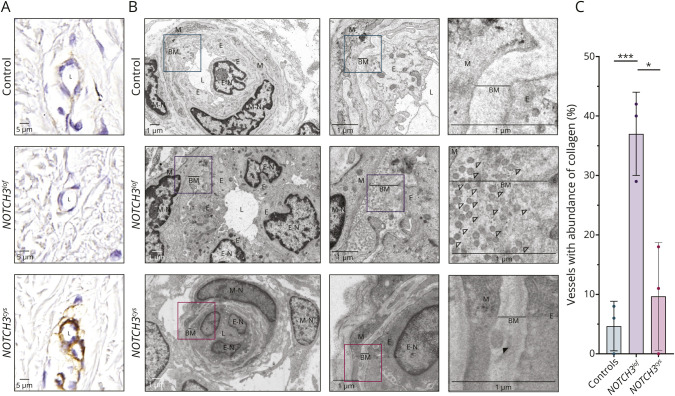
Skin Vessels of *NOTCH3*^*lof*^ Cases Have Increased Deposition of Collagen Fibrils (A) Representative images of *NOTCH3* immunohistochemistry on skin blood vessels in a control, a *NOTCH3*^*lof*^ case (*NOTCH3* c.1321C>T; p.(Arg441*)), and a *NOTCH3*^*cys*^ case (*NOTCH3* c.505C>T; p.(Arg169Cys)). NOTCH3 staining of skin vessels from *NOTCH3*^*lof*^ cases was similar to that in controls while the *NOTCH3*^*cys*^ case shows the typical increased and granular NOTCH3 vessel wall staining of CADASIL. (B) Representative electron microscopy images of a dermal vascular unit of a *NOTCH3*^*lof*^ case showing a thick, amorphous layer of collagen between the endothelial cells and the mural cells (open arrowheads). The image of the *NOTCH3*^*cys*^ case shows the CADASIL-pathognomonic GOM deposits (black arrowhead). Additional images of skin vessels are included in eAppendices 12–13. (C) Bar chart showing the mean percentage of vessels with abundant collagen fibrils in the basal membrane, after qualitative assessment by 2 independent blinded observers (median per patient = 19 vessels, range 5–26). One dot represents 1 patient. On average, over one-third of skin vessels of *NOTCH3*^*lof*^ cases (37%) showed a large quantity of collagen fibrils, which was rarely seen in skin vessels of controls (5%, *p* < 0.001) and *NOTCH3*^*cys*^ cases (10%, *p* = 0.016). BM = basal membrane; CADASIL = cerebral autosomal dominant arteriopathy with subcortical infarcts and leukoencephalopathy; E = endothelial cell; L = lumen; M = mural cell; N = nucleus; *NOTCH3*^*cys*^ = cysteine-altering *NOTCH3*; *NOTCH3*^*lof*^ = *NOTCH3* loss-of-function. Values are significant at ***p < 0.001 and *p < 0.05.

Analysis of skin vessels was performed in 3 siblings from a Dutch family with the *NOTCH3* c.1321C>T; p.(Arg441*) variant (age range 45–49), all of whom had white matter hyperintensities but no other symptoms of small vessel disease (Figure 3, A–D). This showed a negative *NOTCH3* staining (Figure 4A) and absence of GOM (Figure 4B). On electron microscopy, an abundance of collagen fibrils between the mural cells and endothelial cells was seen, forming a thick, amorphous layer in the basal membrane (Figure 4B, eAppendix 12). This abundance of collagen fibrils was observed in over one-third (37%) of the vessels of the *NOTCH3*^*lof*^ cases and was rarely seen in vessels of controls (5%; *p* < 0.001) or in those of *NOTCH3*^*cys*^ cases (10%; *p* = 0.016) (Figure 4C). There was no difference in the presence of electron lucent vacuoles and endothelial protrusions between *NOTCH3*^*lof*^ cases and controls (eAppendix 13).

## Discussion

In this study, we aimed to clarify the small vessel disease phenotype associated with monoallelic *NOTCH3*^*lof*^ variants. By combining data from community-dwelling individuals and clinically ascertained cases with a *NOTCH3*^*lof*^ variant and by histopathologic, neuroimaging, and clinical analyses, we found that monoallelic *NOTCH3*^*lof*^ variants are associated with an increased white matter hyperintensity burden on brain MRI and collagen depositions in the vessel wall. Other small vessel disease neuroimaging markers such as lacunes and microbleeds, and clinical symptoms such as stroke and dementia, were only seen in a minority of cases, predominantly in those with cardiovascular risk factors and at advanced age.

Since the identification of proaggregatory *NOTCH3*^*cys*^ variants as the cause of CADASIL, there has been an ongoing debate about whether *NOTCH3*^*lof*^ variants can also cause CADASIL or a CADASIL-like small vessel disease.^11-16,18,41,42^ In this study, we show that, despite the overlap in the small vessel disease neuroimaging phenotype, there are notable differences regarding vessel wall pathology and clinical and neuroimaging disease expression between *NOTCH3*^*lof*^ and *NOTCH3*^*cys*^ variants. In UK Biobank, the WMHv in *NOTCH3*^*lof*^ cases was comparable to the WMHv in moderate-risk *NOTCH3*^*cys*^ cases; however, in contrast to moderate-risk *NOTCH3*^*cys*^ cases, *NOTCH3*^*lof*^ cases did not have an increased stroke risk. The difference in small vessel disease expression between *NOTCH3*^*lof*^ and *NOTCH3*^*cys*^ variants is likely explained by the differences in the underlying disease pathomechanism, namely reduced *NOTCH3* expression in *NOTCH3*^*lof*^ cases versus NOTCH3 aggregation leading to GOM deposits in cases with a *NOTCH3*^*cys*^ variant.

In line with *NOTCH3*^*lof*^ cases in UK Biobank, most clinically ascertained *NOTCH3*^*lof*^ cases had white matter hyperintensities on brain MRI, but no other small vessel disease markers. Although sample size did not allow for statistical analysis, we observed that in the subset of cases with lacunes or microbleeds, most had cardiovascular risk factors and were older than 60 years. There were a few cases who had a relatively early disease onset and no cardiovascular risk factors. These cases may represent the severe end of the disease spectrum associated with *NOTCH3*^*lof*^ variants, in which the phenotype is aggravated by unknown genetic or environmental modifiers. Given our findings in UK Biobank and the *NOTCH3*^*lof*^ population frequency of 1 in 1,600 individuals worldwide, these cases are probably rare exceptions. In the data set of clinically ascertained *NOTCH3*^*lof*^ cases, ascertainment bias has likely contributed to a higher reported stroke incidence because in the clinical setting, only patients with a CADASIL-like phenotype will receive *NOTCH3* genetic testing. In addition, the genetic testing in these cases was often limited to *NOTCH3* and, therefore, did not include alternative genetic causes of small vessel disease such as *HTRA1*.^43^ In some cases, the diagnosis of stroke was uncertain because there was no neuroimaging evidence of ischemia. On the contrary, in the UK Biobank data set, a healthy volunteer inclusion bias may have led to an underrepresentation of symptomatic individuals^44^ and minor increases in stroke risk may have been missed because of sample size limitations.

Recently, it was discovered that *NOTCH3* expression in brain vascular smooth muscle cells decreases with age and that this reduced expression plays an important role in vascular aging.^2^ We hypothesize that, likewise, in *NOTCH3*^*lof*^ cases, constitutively reduced NOTCH3 protein levels predispose to premature vascular aging. Our observation that *NOTCH3*^*lof*^ cases more frequently had increased collagen deposition in skin vessels is in line with this hypothesis, as vascular aging is associated with an increase in extracellular matrix proteins, including collagen, in the vessel wall.^45,46^ The analysis of skin vessel collagen presented here was performed in 3 cases from a single pedigree and should, therefore, be interpreted with caution. Nevertheless, our findings are in line with a previous report showing an increased expression of collagen in vascular smooth muscle cells of *Notch3*^−/−^ mice^2^ and with reports showing similar collagen depositions in skin vessels of monoallelic and biallelic *NOTCH3*^*lof*^ cases.^3,4^ To what extent vascular collagen deposition occurs in brain vessels of *NOTCH3*^*lof*^ cases, and how this differs from brain vessel collagen deposition observed in small vessel disease due to other etiologies, such as hypertension, is unknown.^47,48^ Future studies, for example in postmortem brain tissue, will be needed to further clarify the brain vessel pathology associated with *NOTCH3*^*lof*^ variants.

If monoallelic *NOTCH3*^*lof*^ variants indeed predispose to premature vascular aging, then these variants may be an unrecognized risk factor of stroke and dementia in the older population, aggravated by cardiovascular risk factors and physiologic vascular aging. At the time of this study, the median age of UK Biobank *NOTCH3*^*lof*^ cases was 58 years and inclusion was limited to age 70 years, such that the effect of *NOTCH3*^*lof*^ variants on the stroke and dementia risk in the older population could not be assessed. The anticipated UK Biobank follow-up will allow for future studies into the clinical consequences of *NOTCH3*^*lof*^ variants in individuals older than 70 years, including reassessment of the cases characterized in this study. Our data suggest that, in line with other (genetic) small vessel diseases, individuals with a *NOTCH3*^*lof*^ variant will benefit from timely and strict cardiovascular risk management.

In contrast to *NOTCH3*^*lof*^ variants, CADASIL-type *NOTCH3*^*cys*^ variants typically do not impair NOTCH3 signaling function.^21,28^ The exceptions are *NOTCH3*^*cys*^ variants located in the ligand-binding domain (EGFr 10 and 11), which likely have both proaggregatory and loss-of-function properties.^29,30^ Recently, *NOTCH3*^*cys*^ variants in EGFr domain 11 were classified as high risk for developing CADASIL, although the NOTCH3 aggregation load associated with these variants was relatively low.^21^ Possibly, in cases with a *NOTCH3*^*cys*^ variant in the ligand-binding domain, the concurrent loss of NOTCH3 signaling acts as a modifier, causing a more severe CADASIL phenotype than would be expected based on the aggregation properties of the *NOTCH3*^*cys*^ variant alone.^29^ Finally, other *NOTCH3* variants causing decreased NOTCH3 function, such as *NOTCH3* c.4810 G>A; (p.Ala1604Thr) and *NOTCH3* c.5926dupC; (p.Leu1976Profs*11), have been reported in patients with small vessel disease.^6,49^ Whether our results can be extrapolated to these *NOTCH3* variants is unknown because it remains to be clarified whether, next to decreased NOTCH3 function, neomorphic effects also play a role in these cases.

In conclusion, we show that the small vessel disease phenotype caused by monoallelic *NOTCH3*^*lof*^ variants is characterized by increased vessel wall collagen deposition and white matter hyperintensities on brain MRI. The small vessel disease remains subclinical in most cases but may be exacerbated by cardiovascular risk factors and aging. Our results contribute to improved counseling and management of individuals in whom a *NOTCH3* loss-of-function variant is found and suggest that *NOTCH3* loss-of-function variants, which occur in 1 in 1,600 individuals worldwide, may be an unrecognized genetic risk factor of stroke and dementia in the older population.

## Glossary

CADASIL: cerebral autosomal dominant arteriopathy with subcortical infarcts and leukoencephalopathy
EGFr: epidermal growth factor-like repeat
FLAIR: fluid-attenuated inversion recovery
gnomAD: Genome Aggregation Database
GOM: granular osmiophilic material
ICD-9: International Classification of Diseases, Ninth Revision
ICD-10: International Classification of Diseases, 10th Revision
IQR: interquartile range
*NOTCH3* ^*cys*^: cysteine-altering *NOTCH3*
*NOTCH3* ^*lof*^: *NOTCH3* loss-of-function
nWMHv: normalized WMHv
OR: odds ratio
PSMD: peak width of skeletonized mean diffusivity
PVS: perivascular space
WMH: white matter hyperintensity
WMHv: WMH volume
